# Mycotoxins and Mycotoxigenic Fungi in Poultry Feed for Food-Producing Animals

**DOI:** 10.1155/2014/968215

**Published:** 2014-07-08

**Authors:** Mariana Vanesa Greco, María Luisa Franchi, Silvia Laura Rico Golba, Alejandro Guillermo Pardo, Graciela Noemí Pose

**Affiliations:** ^1^Departamento de Ciencia y Tecnología, Universidad Nacional de Quilmes (UNQ), Roque Sáenz Peña 352, B1876BXD Bernal, Provincia de Buenos Aires, Argentina; ^2^Consejo Nacional de Investigaciones Científicas y Técnicas (CONICET), Avenida Rivadavia 1917, C1033AAJ CABA, Argentina; ^3^Universidad Nacional de Río Negro (UNRN), Mitre 331, 8336 Villa Regina, Río Negro, Argentina; ^4^Facultad de Ciencias Veterinarias, Universidad Nacional de La Plata, Calle 60 y 118 S/N, 1900 La Plata, Buenos Aires, Argentina

## Abstract

Moulds are capable of reducing the nutritional value of feedstuff as well as elaborating several mycotoxins. Mycotoxin-contaminated feed has adverse effects on animal health and productivity. Also, mycotoxins may be carried over into meat and eggs when poultry are fed with contaminated feed. In a point prevalence study feedstuff used for poultry nutrition in Argentina was analyzed for fungal flora, natural incidence of selected mycotoxins, and nutritional quality. Ten mould genera were recovered, six of them known to be mycotoxigenic. More than 28 species were determined. Fumonisins were detected in all the samples (median 1,750 ppb). Forty-four out of 49 samples (90%) were contaminated with DON (median 222 ppb) and OTA (median 5 ppb). Also, 44 out of 49 samples were contaminated with aflatoxins (median 2.685 ppb), 42 samples (86%) with ZEA (median 50 ppb), and 38 samples (78%) with T2-toxin (median 50 ppb). Ninety percent of the samples had at least one type of nutritional deficiency. This study indicates the need for continuous assessment of the mycological status of animal feed production, in order to feed animals for optimal performance ensuring food safety.

## 1. Introduction

The rapid growth in the meat sector has been underpinned by the rising demand of poultry meat, which has consistently increased about threefold the rate of population growth over each of the past five decades [1].

The presence of microscopic fungi affects the quality of feeds, their organoleptic attributes, and nutritional quality [2]. Moulds like other microorganisms will assimilate and utilize the most readily available nutrients in the materials they grow upon and spoilage may result in the loss of 5 to 100% of the nutrients in the feed [3]. Regarding nutritional quality, lipids, proteins, and minerals are of essential importance for the proper development and growth of farm animals. The quantity and the nutritional requirements of feed depend on the weight and age of the poultry as well as the season. Healthy poultry require sufficient amounts of carbohydrates, lipids, and proteins, along with the necessary vitamins and dietary minerals [4, 5].

In addition to their negative impact on nutritional and organoleptic properties, moulds can synthesize different mycotoxins [6]. It is well known that contamination of animal feed with mycotoxins may induce sanitary disturbances and mortality among animals and secondary contamination of human consumers via eggs, meat, or milk [7]. Consumption of a mycotoxin-contaminated diet may induce acute and long-term chronic toxic effects [8, 9]. With respect to humans and animals, in general terms, mycotoxins exhibit toxic actions and are characterized by carcinogenic, mutagenic, teratogenic, and estrogenic properties [2].

Most toxic species belong to the genera* Aspergillus, Penicillium, Fusarium,* and* Alternaria* [10]. According to several authors mycotoxins such as aflatoxins, zearalenone, T2-toxin, deoxynivalenol, ochratoxin A, fumonisins, and patulin can be considered the most common mycotoxins found in feed and food [11–13].

Most mycotoxicoses of poultry are caused by an intake of low concentration of contaminants over a long whit the typical chronic symptoms of poor growth, poor feed efficiency, and suboptimal production. Ingestion of higher concentration however leads to acute clinical symptoms associated with specific vital organs, the immune system, and other aspects of avian physiology as well as mortality [2, 14].

For quality control the identification of the contaminating mycobiota is essential because it provides data on the potential production of its mycotoxins and is a helpful indicator to determine feed hygienic quality [15].

In the European Union the limits for several mycotoxins are regulated. The maximum content of aflatoxin B1 in poultry feed has been set in 0.02–0.05 ppm. Moreover, based on studies about feed to food mycotoxin transfer limits for ochratoxin A, deoxynivalenol, zearalenone, and fumonisins B1 and B2 in feed have been recommended [16–18].

In Argentina there is information on the fungal status including mycobiota and mycotoxins in poultry feed, but this is limited to the central region of the country, particularly Córdoba province and more recently to Entre Ríos province [19–25]. In Argentina three geographical regions can be clearly distinguished: the northern-central with grasslands, the southern (Patagonia) with tablelands, and the western with mountains.

The aim of the present study was to determine the hygienic and nutritional quality and the occurrence of selected groups of fungi and mycotoxins in poultry feeds in other important producing regions of Argentina, not previously studied. Samples were collected from the central region, particularly from Buenos Aires (the largest producing area of Argentina) and La Pampa provinces, and from Río Negro province (an increasing producing area belonging to the Patagonia region). The study includes enumeration and identification of mould genera and species, natural levels of mycotoxins such as aflatoxin, ochratoxin A (OTA), T2-toxin, fumonisin, deoxinivalenol (DON), and zearalenone (ZEA), and the evaluation of some variables related to nutritional quality (crude proteins, fats, humidity, and total ashes).

## 2. Materials and Methods

### 2.1. Samples

A total of 49 representative samples (1-2 kg per sample) of nine commercially prepared and farm mixed poultry feeds were collected from Buenos Aires, La Pampa, and Río Negro provinces during 2010 (details about samples are shown in Table 1). All samples were homogenized and divided to obtain a 1 kg of working sample for analysis. Each sample was ground in a laboratory mill.

The main component of samples was corn followed by deactivated soybean, soybean pellet, wheat bran, sorghum, animal proteins, and grain mill products.

For mycological examination feed samples were immediately analyzed upon arrival or they were stored for 2-3 days in paper bags at room temperature (about 25°C). Feed samples intended for mycotoxin analysis were stored at −20°C.

### 2.2. Mycobiota Determination

The dilute plate technique was used for enumeration and isolation of fungi [10]. Ten grams of each milled feed sample was mixed with 90 mL of 0.1% peptone and shaken on a horizontal shaker for 20 minutes. Then, 0.1 mL of this dilution was inoculated on each of three different media: Dichloran Rose Bengal Chloramphenicol Agar (DRBC) to enumerate total culturable fungi, Dichloran 18% Glycerol Agar (DG18) to enumerate xerophilic fungi, and Dichloran Chloramphenicol Peptone Agar (DCPA) for selective isolation of* Alternaria *and* Fusarium *species. Plates were incubated at 25°C for 7 days. The DCPA plates were incubated under 12 hours of light: 12 hours of darkness photoperiod. For counting, plates containing 10–100 colonies were used and the results were expressed as colony-forming units per gram of sample (CFU/g) [10]. Individual CFU/g counts for each colony type, considered to be different, were recorded. Representative colonies of each type were transferred for subculturing onto plates with Malt Extract Agar (MEA) or Water Agar (WA), for moulds suspected to belong to* Alternaria* or* Fusarium* genera. Filamentous fungi were identified at genus level according to macro- and microscopic criteria in accordance with Samson et al. [26]. Fungal isolates were identified at species level:* Penicillium* and* Aspergillus *spp. according to Pitt and Hocking [10],* Fusarium *spp. according to Nelson et al. [27],* Alternaria *spp. according to Simmons [28], and other fungi according to Pitt and Hocking [10]. The isolation frequency (Fr) and relative density (RD) of genus/species were calculated according to Gonzalez et al. [29], Pacin et al. [30], and Saleemi et al. [31] as follows:

(1) Fr(%)=number  of  samples  with  a  genus  or  speciestotal  number  of  samples×100,RD(%)=number  of  isolates  of  a  genus  or  speciestotal  number  of  fungi  isolated×100.

*Aspergillus *and* Penicillium *isolates were preserved on agar slants of MEA and* Alternaria *and* Fusarium *isolates on Potato Dextrose Agar (PDA) at 4°C and cryopreserved in 18% glycerol at −20°C.

### 2.3. Mycotoxin Analysis

To evaluate mycotoxin occurrence, feed samples were subjected to quantitative analysis using ELISA-based analytical test kits for aflatoxin, ochratoxin A, T2-toxin, fumonisin, deoxynivalenol, and zearalenone (RIDASCREEN FAST, R-BiopharmAG). The extraction procedures were according to manufacturer's protocol. In brief, 5 g of ground sample was extracted with 25 mL of 70% methanol for aflatoxins, T2-toxin, ZEA, and fumonisins. For OTA and DON, samples were extracted with 12.5 mL of 70% methanol or 100 mL of distilled water, respectively. Afterwards, samples were shaken vigorously for 3 minutes and the extracts filtered through Whatman N°1 paper. Then, aflatoxins, OTA, T2-toxin, and ZEA filtrates were diluted with distilled water in the ratio 1 : 1 and fumonisin filtrates in the ratio 1 : 14. Fifty *μ*L of the diluted filtrate per well was used for testing [32].

### 2.4. Nutritional Analysis

Feed samples were subjected to proximate analysis in accordance with standard methods described by the AOAC [33]. In terms of nutritional composition crude proteins (Kjeldahl) (Method 984.13), fats (Soxhlet) (Method 945.16), humidity (Method 934.01), and total ashes (to constant weight) (Method 942.05) were determined. Prior to analysis feed samples supplied as pellets were ground with a blade grinder or laboratory mill. All analyses were done in duplicate.

To obtain the moisture content the oven-drying method was used. Samples were dried overnight in an oven at 100°C, cooled in a desiccator to room temperature, and weighed. This procedure was performed as many times as necessary until constant weight. The result was obtained by weight difference with the sample at the beginning of the procedure.

All other analyses were performed on dry matter.

Determination of total ashes (mineral content) was performed by ignition of samples in a muffle oven set at 600°C until complete calcination of organic components. This is achieved when ashes obtained are white or light gray and the weight is constant. Samples were cooled in a desiccator at room temperature and weighed. At the beginning of this procedure samples were heated until obtention carbonaceous residue.

A Soxhlet extractor was used to determine free lipid content. Dried samples were weighed into an extraction thimble. The extraction was performed using petroleum ether of 60–80 boiling range for approximately 2 hours (occurring at least 7 cycles of filling and siphoning of the extraction tube). Then the flask with the solvent-free lipid extract was dried in an oven, cooled in a desiccator, and weighed. Results are expressed as percentage of lipids.

Determination of total nitrogen (crude protein) was conducted using the Kjeldahl method. The percentage of crude protein was calculated by multiplying the total nitrogen by a correction factor of 6.25.

### 2.5. Statistical Analysis

Fungal counts and toxins content were analyzed with the Kruskal-Wallis test. Multiple Range Test for variables was employed to compare means of fungal counts and toxins content of samples from different provinces. Differences were considered significant when *P* < 0.05. Statgraphics Centurion XVI version 16.1.18 was used for all analyses.

## 3. Results and Discussion

Fungal contamination of animal feed, with the consequent mycotoxin production, is one of the major threats to human and animal health [34]. In our study, all the samples analyzed were contaminated with fungi and mycotoxins.

Fungal counts (CFU/g) were obtained from different poultry feed samples on the three culture media and the mycological quality was determined according to Gimeno [35]: samples can be qualified as good (count range < 3.10^4^ UFC/g), regular (count range 3.10^4^–7.10^4^ UFC/g), and bad (>7.10^4^ UFC/g). On both media, DRBC and DCPA, fungal count range was 10–10^6^ CFU/g. According to the mycological quality criterion 56% (26/46) samples could be qualified as good, 7% (3/46) as regular, and 37% (17/46) as bad (on DRBC). In Argentina there is information about the mycobiota and natural occurrence of mycotoxins in feedstuff [19–24, 32, 36, 37]. However these reports on poultry feed refer mostly to Córdoba and Entre Rios provinces (central area of the country). With respect to the hygienic quality of poultry feed the last report from Córdoba province [24] was informed that 79% of finisher poultry feed samples exceeded the maximum allowed total fungal count to ensure hygienic quality. In Entre Rios province, 120 pelleted poultry feed samples were analyzed and total fungal count was below the established value (1 × 10^4^ UFC/g) [25]. Significant differences among fungal contamination levels in feed from Río Negro province were observed (*P* < 0.05) while nonsignificant differences were found between feed samples from each province. These results indicate the need for continuous assessment of the mycological status of animal feed production.

Xerophilic fungal counts were in the range 1 × 10^2^–1.45 × 10^6^ CFU/g. Spoilage of feedstuff can be due to xerophilic fungi, which are capable of rapid growth above about 0.77 water activity (*a*_*w*_) and of slow growth at 0.75 *a*_*w*_ and below-down to about 0.68 *a*_*w*_ [10]. Although *a*_*w*_ of poultry feed is low (average 0.537) it should be kept in a dry and fresh place. If by carelessness or negligence feed is not kept under proper conditions, as the humidity increases fungal growth and proliferation of xerophilic fungi can occur. Fungal counts average and median were similar between all culture media tested (10^5^ CFU/g and 10^3^ CFU/g, resp., data not shown).

The mycobiota and natural incidence of aflatoxin B1, ZEA, and DON were investigated by Dalcero and colleagues [19, 20]. In the first study* Penicillium* (98%),* Fusarium* (87%), and* Aspergillus* (52%) were the most prevalent genera determined whereas in the second study* Aspergillus* (85%) and* Fusarium *(70%) were the dominant genera. The predominant species of the genera abovementioned were particularly studied [21, 22]. The predominant species of* Aspergillus* were* A. flavus *and* A. parasiticus*. For* Penicillium *they were* P. brevicompactum, P. purpurogenum, *and* P. oxalicum*. With regard to* Fusarium *genus the predominant species were* F. moniliforme *and* F. nygamai*. Aflatoxin B1 was the most prevalent mycotoxin. The last study [24] indicates that* A. flavus* was the only species belonging to section* Flavi* which was isolated while* Fusarium verticillioides* was the prevalent species. In Entre Rios province* A. flavus* and* A. parasiticus* were the aflatoxigenic species isolated.

In our study ten mould genera were recovered, four of them known to be mycotoxigenic [10]. The frequency (Fr) and relative density (RD) are showed in Table 2. While the predominant genera are coincident with the abovementioned results, the most frequent mycotoxigenic fungi were those from the genus* Fusarium* (69.6), followed by* Eurotium* (52.2%),* Penicillium* (45.65%), and* Aspergillus* (43.5%), although the presence of* Eurotium* spp. was not previously reported. The occurrence of* Aspergillus* spp. and* Fusarium* spp. is widespread in tropical countries, also, in corn in Argentina [38]. Corn is the main component of poultry feed in the samples analyzed.

More than 28 species were recovered from poultry feed samples. Several species from different genera were determined in each sample. Up to 12 different species were recovered in several samples. The frequency (Fr) and relative density (RD) are showed in Table 3. Different to the studies from Cordoba province, the most prevalent mycotoxigenic* Fusarium* species determined was* F. proliferatum* (Fr 69.5%–RD 17.7%). In accordance with previous studies in Córdoba [19, 20] and Entre Rios provinces [25],* A. flavus* and* A. parasiticus *(Fr 6.5%) were the aflatoxigenic species isolated*. Penicillium chrysogenum *and* P. nalgiovense* (Fr 13.0%) were the most prevalent* Penicillium* species recovered. Some other studies have shown that most poultry feeds are contaminated with species belonging to* Penicillium, Fusarium, *and* Aspergillus* genera [23]. From 120 samples of poultry feed Magnoli et al. [23] reported the presence of 15 genera of filamentous fungi.* Fusarium *and* Penicillium* were isolated in 67.5% of the samples and* Aspergillus* in 57.5% of them [23]. In South America, mycobiota and natural occurrence of aflatoxins, fumonisins, and ZEA in poultry feed were studied in Rio de Janeiro, Brazil [39]. The most frequent genus isolated was* Penicillium *(41.26%) followed by* Aspergillus* (33.33%) and* Fusarium* (20.63%).* Penicillium citrinum, A. flavus F. verticillioides,* and* F. graminearum *were the most prevalent species in decreasing order. On the other hand, we found* Fusarium* and* Penicillium* species, followed by* Aspergillus*, as prevalent mycobiota (Tables 2 and 3).* Eurotium *species such as* E. amstelodami, E. repens* (Fr 26.1%),* E. chevalieri* (Fr 19.6%), and* E. rubrum* (15.2%) were also found.

Table 4 shows the distribution concentration of mycotoxins in the poultry feed tested in this study. Fumonisins were detected in all the samples analyzed in a range of 222–6,000 ppb (median 1,750 ppb). Forty-four out of 49 samples (90%) were contaminated with DON (median 222 ppb) and OTA (median 5 ppb). Also, 44 out of 49 samples were contaminated with aflatoxins (median 2.685 ppb), 42 samples (86%) were contaminated with ZEA (median 50 ppb), and 38 samples (78%) were contaminated with T2-toxin (median 50 ppb). Fumonisins and DON showed the highest levels detected (6,000 ppb and 318 ppb, resp.). Significant differences were found for fumonisins in feed from La Pampa, for OTA and T2-toxin in feed from Río Negro, and for ZEA in feed from Buenos Aires. However, no differences were found for aflatoxins (*P* = 0.0604) and DON (*P* = 0.1211).

Magnoli et al. [23] also found that fumonisins had the highest incidence and were found in 97% of the analyzed samples followed by aflatoxin B1 (46%), ZEA (18%), and DON (6%). Jaramillo [40] found that fumonisin B1 and ZEA occurred in most of the samples, whereas aflatoxin B1 occurred in lower proportion. Aflatoxin B1 values ranged between 1.2 and 17.5 ppb. Fumonisin B1 levels ranged between 1,500 and 5,500 ppb. Zearalenone levels ranged between 100 and 7,000 ppb. In both reports, the simultaneous occurrence of two carcinogenic mycotoxins, aflatoxin B1 and fumonisin B1, was determined in feed intended for poultry consumption. In Córdoba province several mycotoxins were monitored in feeds. One hundred percent of poultry samples were contaminated with fumonisins B1, but aflatoxin B1 and T2- toxin, in between other selected mycotoxins, were not detected in any poultry feed [24]. On the other hand, cooccurrence of fumonisin B1, T2-toxin, and HT-2 was detected in 100% of the samples of poultry feed analyzed in Entre Rios province, with mean levels from 4502 to 5813, 19.6 to 30.3, and 6.7 to 21.6 ppb, respectively. Also, samples were cocontaminated with aflatoxin B1 [25].

In this work cooccurrence of mycotoxins was determined in all of the samples analyzed. Cooccurrence of at least three out of six mycotoxins was determined in all of the samples analyzed. Cooccurrence of six mycotoxins was determined in 57% (28/49) of the samples. The combined action of mycotoxins can generate an interactive effect such as additivity, synergism, or antagonism. The synergistic interaction causes the most toxic effects in the case of aflatoxins and OTA, or aflatoxins and toxin T2. An additive effect of aflatoxins and DON in fattening poultry has been demonstrated. This effect was obvious in proventriculus weight, blood glucose level, and lactate dehidrogenase activity. Synergistic effects in fattening poultry were observed in the case of aflatoxins and OTA. This interaction provoked reduction of body weight and increased mortality. Moreover, synergistic effects were observed for aflatoxins and toxin T2. A reduction in body weight, increased relative weight of kidneys, gizzard and hearth, and reduction of the mean corpuscular volume and potassium levels in blood were observed [41]. In our study cooccurrence of aflatoxins and OTA was observed in 42 out of 49 samples. The cooccurrence of aflatoxins and T2-toxin was observed in 37 samples. Synergism between aflatoxins and toxin T2 is extremely important in poultry due to the prevalence and severity of both mycotoxins [40]. Simultaneous occurrence of two carcinogenic mycotoxins, aflatoxins and fumonisins, was recorded in 44/49 (90%) of the samples.

Around the world other researchers have studied the mycobiota and mycotoxins in poultry feed during the last years. Okoli et al. [3] analyzed the mycobiota of commercial poultry feed in Nigeria. The common moulds isolated were* Aspergillus *spp.*, Penicillium *spp.*, Mucor *spp.*, Rhizopus *spp.*, Epicoccum *spp.*, Gymnoascus *spp.*, Cladosporium *spp.*, Mortierella *spp., yeast, and bacteria. In Pakistan, Saleemi et al. [31] found* Aspergillus* species as most predominant fungi, followed by* Penicillium, Fusarium,* and* Alternaria*. Among the* Aspergillus* isolates,* A. niger* (37.74%) was the most frequently isolated species followed by* A. flavus* (22.64),* A. ochraceous* (16.98%),* A. parasiticus* (13.21%),* A. carbonarius* (3.77%),* A. fumigatus* (3.77%), and* A. oryzae* (1.89%). The proportion of toxigenic fungi among* Aspergillus *isolates was 73.58%. Shareef [6] recovered fourteen mould genera from Iraq poultry feed. The most frequent fungi were* Aspergillus* followed by* Penicillium, Mucor, Rhizopus, Scopulariopsis, Alternaria, *and* Eurotium*. They also study the presence of 4 mycotoxins; aflatoxins and ochratoxins were determined in 91.1% of the samples, fumonisins were determined in 51.1%, and T2-toxin was determined in 2.2% of the samples [3, 6, 31].

The toxicogenic fungal contamination of the raw materials occurs during the preharvest and/or the postharvest periods, and the finished feeds are exposed during production, processing, transportation, and storage [24]. It has been observed that moulds and mycotoxins presence varies depending on the geographical location and the year. Temperature and humidity play important roles not only in the development of fungi but also in mycotoxin production [24].

With respect to nutritional aspects, poor health can be caused by an imbalance of nutrients either by deficiency or by excess. Also, malnutrition if prolonged eventually leads to animal death. We found that 90% of the analyzed samples presented some kind of nutritional deficiency. The results are showed in Table 5. Forty-one out of 42 samples contained adequate moisture. However, 90% of them had at least one type of nutritional deficiency. Nineteen out of 38 samples did not reach the minimum of protein content required, 31 out of 40 did not contain the minimum requirements of lipids, and 9 out of 38 exceeded the ash content.

This information is quite important considering poultry health, and hence productivity requires proper supply of these nutrients. The nutritional quality of chicken meat lies in their protein, iron, zinc, phosphorus, niacin, folic acid, and vitamins A, B6, and B12 content. The nutritional quality of the chicken thus depends on their feeding conditions.

Moulds are capable of reducing the nutritional values of feedstuff. When conditions are optimal for moulds to colonize grains in the field or in storage, their first effect is utilization of nutrients for their metabolism and propagation. This results in decreased nutritional value of feeds. Energy, crude protein, and crude fat values of mouldy corn were decreased by 5, 7, and 63%, respectively [41].

## 4. Conclusions

In this work, although the amounts of the mycotoxins detected on poultry feed were lower than the regulation limits established, the cooccurrence of several mycotoxins was demonstrated. The scientific literature offers a broad spectrum of information on the effects of individual mycotoxins in various animal species but concurrent exposure to multiple mycotoxins is more likely in feedstuff [8]. While mycotoxins have attracted worldwide attention due to their direct impact on human health, due to food contamination, it is also relevant to consider to what extent mycotoxins can be carried over into edible tissues like meat and eggs when poultry are fed with contaminated feed.

We suggest that periodic monitoring and the application of the Hazard Analysis and Critical Control Points (HACCP) on the prevention and control of mycotoxins in the animal feed industry are needed in order to guarantee the hygienic and nutritional quality of feed to ensure health and productivity of poultry as well as prevent human foodborne diseases.

## Figures and Tables

**Table 1:** Characteristics of the samples analyzed.

Province	Type of feed	Number of samples	Sample characteristics	
Bs. As.	Broiler Chicken Starter	1	Commercial	Pelleted
	Broiler Chicken Starter	1	Noncommercial, private use	Milled
	Pullet Starter	4	Commercial	Pelleted
	Pullet Finisher	4	Commercial	Pelleted
	Laying Hen Finisher	3	Commercial	Pelleted
	Choique	5	Commercial	Pelleted

La Pampa	Pullet Starter	4	Commercial	Pelleted
	Pullet Finisher	4	Commercial	Pelleted
	Laying Hen Finisher	2	Commercial	Pelleted

Río Negro	Broiler Chicken Pre Starter	1	Commercial	Milled
	Broiler Chicken Starter	2	Commercial	Milled
	Broiler Chicken Starter	3	Noncommercial, private use	Milled
	Broiler Chicken Finisher	1	Commercial	Pelleted
	Broiler Chicken Finisher	3	Noncommercial, private use	Milled
	Pullet Starter	1	Commercial	Pelleted
	Pullet Finisher	1	Commercial	Pelleted
	Laying Hen Starter	1	Commercial	Pelleted
	Laying Hen Starter	1	Noncommercial, private use	Milled
	Laying Hen Finisher	1	Commercial	Milled
	Laying Hen Finisher	3	Noncommercial, private use	Milled

**Table 2:** Fungal genus present in poultry feed samples.

Genus	Number of isolates	Fr (%)∗	RD (%)∗∗
*Aspergillus *	20	43.5	10.5
*Cladosporium *	22	47.8	11.5
*Eurotium *	24	52.2	12.5
*Fusarium *	32	69.6	16.7
*Mucor *	18	39.1	9.4
*Penicillium *	21	45.6	10.9
*Paecilomyces *	1	2.2	0.5
*Scopulariopsis *	2	4.3	1.0
Others	16	34.8	8.4
Yeast	36	78.3	18.8

*Isolation frequency.

∗∗Isolation relative density.

**Table 3:** Fungal species present in poultry feed samples.

Species	Number of isolates	Fr (%)∗	RD (%)∗∗
*Eurotium amstelodami *	12	26.1	6.6
*E. chevalieri *	9	19.6	4.9
*E. herbariorum *	1	2.2	0.5
*E. repens *	12	26.1	6.6
*E. rubrum *	7	15.2	3.9
*Eurotium *sp.	2	4.3	1.1
*Aspergillus candidus *	4	8.7	2.2
*A. flavus *	3	6.5	1.7
*A. fumigatus *	1	2.2	0.5
*A. niger *	3	6.5	1.7
*A. niveus *	1	2.2	0.5
*A. parasiticus *	3	6.5	1.7
*A. terreus *	2	4.3	1.1
*A. versicolor *	1	2.1	0.5
*Aspergillus *sp.	7	15.2	3.9
*Cladosporium cladosporioides *	22	47.8	12.1
*Penicillium brevicompactum *	1	2.2	0.5
*P. chrysogenum *	6	13.0	3.3
*P. citrinum *	1	2.2	0.5
*P. decumbens *	1	2.2	0.5
*P. funiculosum *	3	6.5	1.7
*P. implicatum *	3	6.5	1.7
*P. nalgiovense *	6	13.0	3.3
*P. oxalicum *	2	4.3	1.1
*P. pinophilum *	2	4.3	1.1
*P. variabile *	1	2.2	0.5
*Penicillium *sp.	6	13.0	3.3
*Fusarium proliferatum *	32	69.5	17.7
*Fusarium *sp.	5	10.9	2.8
*Paecilomyces variotii *	1	2.2	0.5
*Scopulariopsis brevicaulis *	1	2.2	0.5
*S. fusca *	1	2.2	0.5
*Scopulariopsis *sp.	1	2.2	0.5
*Mucor *sp.	18	39.1	9.9

*Isolation frequency.

∗∗Isolation relative density.

**Table 4:** Concentration of different mycotoxins in poultry feed samples (ND: not detected).

Province	Parameter	Mycotoxins (ppb)					
		Aflatoxin∗	DON∗	Fumonisin∗	OTA∗	T2-toxin∗	ZEA∗
Bs. As.	Number of samples positive/tested	17/18 (94.5%)	18/18 (100%)	18/18 (100%)	17/18 (94.5%)	15/18 (83.4%)	12/18 (66.7%)
	Minimum	ND	222.00	222.00	ND	ND	ND
	Maximum	11.85	318.00	4980.00	12.31	50.00	50.00
	Median	4.32	222.00	890.00	5.00	50.00	50.00

La Pampa	Number of samples positive/tested	8/10 (80%)	8/10 (80%)	10/10 (100%)	9/10 (90%)	4/10 (40%)	9/10 (90%)
	Minimum	ND	ND	1160.00	ND	ND	ND
	Maximum	4.24	247.00	6000.00	5.00	50.00	95.58
	Median	1.96	222.00	4565.00	5.00	ND	50.00

Río Negro	Number of samples positive/tested	17/18 (94.5%)	18/18 (100%)	18/18 (100%)	18/18 (100%)	18/18 (100%)	18/18 (100%)
	Minimum	ND	222.00	222.00	5.00	ND	50.00
	Maximum	37.67	222.00	6000.00	13.20	107.23	75.76
	Median	2.47	222.00	708.50	5.00	50.00	50.00

*LOD (ppb): aflatoxin: <1.7; DON: 200; fumonisins: 222; OTA: 5; T2-toxin: <20; ZEA: 17–41.

**Table 5:** Nutritional composition of poultry feed samples analyzed and recommendations (NA: not analyzed).

Animal/type of animal feed	Sample	Nutritional analysis			
		Moisture	Proteins	Lipids	Ashes
Broiler Chicken Prestarter	P33	10,29	21,34	3,65	5,89

*Recommendations: *	Protein (min) = 22%				
	Lipids (min) = 5–5,5%				
	Ashes (max) = 6–8%				
	Moisture (max) = 12%				

Broiler Chicken Starter	P1	12,88	NA	NA	NA
	P18	11,75	NA	NA	NA
	P32	8,98	22,99	1,78	6,62
	P34	9,08	24,12	2,48	6,99
	P35	12,04	19,85	2,49	5,26
	P42	Error	23,66	5,31	5,44
	P45	10,73	21,13	4,55	6,15

*Recommendations: *	Protein (min) = 20–22%				
	Lipids (min) = 3–5%				
	Ashes (max) = 4.9–8%				
	Moisture (max) = 12-13%				

Pullet Starter	P12	10,21	22,21	2,89	6,32
	P8	10,20	10,58	1,92	9,67
	P13	9,48	19,56	4,39	11,45
	P16	9,10	10,46	1,79	10,63
	P19	Error	21,11	4,97	5,46
	P21	NA	NA	NA	NA
	P24	11,00	NA	2,38	7,97
	P28	10,35	21,48	2,68	5,80
	P39	9,99	18,82	4,94	5,01

*Recommendations: *	Protein (min) = 20%				
	Lipids (min) = 6%				
	Ashes (max) = —				
	Moisture (max) = 12.5%				

Broiler Chicken Finisher	P29	11,54	NA	NA	NA
	P36	12,75	17,88	2,08	4,78
	P38	9,47	19,22	2,66	6,29
	P43	11,63	16,93	4,30	5,38

*Recommendations: *	Protein (min) = 18–18.5%				
	Lipids (min) = 4-5%				
	Ashes (max) = 7.5–8%				
	Moisture (max) = 12%				

Pullet Finisher	P9	7,61	11,60	4,26	NA
	P10	7,58	18,54	8,08	8,64
	P15	9,64	19,46	8,90	5,66
	P17	7,44	10,78	3,61	10,20
	P20	10,61	20,81	4,99	6,41
	P22	10,18	18,35	6,16	8,50
	P27	7,37	20,26	8,59	6,53
	P25	10,77	NA	2,70	NA
	P40	9,44	19,03	5,27	4,74

*Recommendations: *	Protein (min) = 19.5%				
	Lipids (min) = 5%				
	Ashes (max) = —				
	Moisture (max) = 12.5%				

Laying Hen Starter	P31	8,94	19,06	3,25	17,28
	P46	11,85	20,72	4,69	9,03

*Recommendations: *	Protein (min.) = 20%				
	Lipids (min) = 6%				
	Ashes (max) = 5%				
	Moisture (max) = 12%				

Laying Hen Finisher	P7	8,63	14,10	3,25	11,29
	P11	8,08	13,36	3,21	15,89
	P14	8,86	18,86	3,25	12,42
	P23	10,01	13,38	3,89	14,00
	P26	NA	NA	NA	NA
	P30	10,18	NA	NA	NA
	P37	11,34	18,82	2,33	10,37
	P41	11,34	18,82	2,33	10,37
	P44	8,83	12,21	3,91	9,51

*Recommendations: *	Protein (min) = 16%				
	Lipids (min) = 6%				
	Ashes (max) = 5%				
	Moisture (max) = 12%				

Choique (*Pterocnemia pennata*)	P2	9,60	22,01	3,22	10,07
	P3	9,85	21,69	3,60	10,45
	P4	9,20	21,94	3,29	10,02
	P5	8,97	21,97	3,25	10,04
	P6	9,75	21,97	3,25	10,04

*Recommendations: *	Protein (min) = 20%				
	Lipids (min) = 5%				
	Ashes (max) = 11%				
	Moisture (max) = 12%				

Recommendations values were obtained from producers.
